# Frequency and co-prescription pattern of Chinese herbal products for hypertension in Taiwan: a Cohort study

**DOI:** 10.1186/s12906-015-0690-8

**Published:** 2015-06-06

**Authors:** Pei-Rung Yang, Wei-Tai Shih, Yen-Hua Chu, Pau-Chung Chen, Ching-Yuan Wu

**Affiliations:** Department of Traditional Chinese Medicine, Chang Gung Memorial Hospital, No.6, W. Sec., Jiapu RD., Puzi City, Chia-Yi County 61363 Taiwan; Institute of Occupational Medicine and Industrial Hygiene, National Taiwan University College of Public Health, Taipei, 10055 Taiwan; Department of Public Health, National Taiwan University College of Public Health, Taipei, 10055 Taiwan; Department of Environmental and Occupational Medicine, National Taiwan University Hospital and National Taiwan University College of Medicine, Taipei, 10055 Taiwan; Graduate Institute of Clinical Medical Sciences, Chang Gung University, College of Medicine, Kaohsiung, 83301 Taiwan; Kidney and Diabetic Complications Research Team, Chang Gung Memorial Hospital, Chia-Yi County, 61363 Taiwan

**Keywords:** Complementary and alternative medicine, Traditional Chinese products, Hypertension, National Health Insurance Research Database, Traditional Chinese medicine

## Abstract

**Background:**

Chinese herbal products (CHPs) have been frequently used among patients with chronic diseases including hypertension; however, the co-prescription pattern of herbal formulae and single herbs remain uncharacterized. Thus, this large-scale pharmacoepidemiological study evaluated the frequency and co-prescription pattern of CHPs for treating hypertension in Taiwan from 2003 to 2009.

**Methods:**

The database of traditional Chinese medicine (TCM) outpatient claims was obtained from the National Health Insurance in Taiwan. Patients with hypertension during study period were defined according to diagnostic codes in the International Classification of Disease Ninth Revision, Clinical Modification. The frequencies and percentages of herbal formula and single herb prescriptions for hypertension were analyzed. We also applied association rules to evaluate the CHPs co-prescription patterns.

**Results:**

The hypertension cohort included 154,083 patients, 123,240 patients of which (approximately 80 %) had used TCM at least once. In total, 81,582 visits involving CHP prescriptions were hypertension related; Tian-Ma-Gou-Teng-Yin and Dan Shen *(Radix Salvia Miltiorrhizae)* were the most frequently prescribed herbal formula and single herb, respectively, for treating hypertension.

**Conclusions:**

This study elucidated the utilization pattern of CHPs for treating hypertension. Future studies on the efficacy and safety of these CHPs and on drug–herb interactions are warranted.

## Background

Hypertension, a major medical and public health concern affecting approximately 972 million adults worldwide, is the main risk factor for cardiovascular, brain, and kidney diseases [1]. In addition, hypertension is consistently accompanied by other metabolic diseases, such as diabetes mellitus and lipid abnormalities, the incidence of which has increased recently [2, 3]. Moreover, hypertension causes a loss of 92 million disability adjusted life years and is responsible for 7.6 million excess deaths yearly [4]. Thus, antihypertensive drugs are primarily used to control blood pressure, thereby preventing the occurrence or progression of cardiovascular diseases and other complications. Despite the prevalence of hypertension and its associated complications, the control of this disease is inadequate. Although several antihypertensive drugs, such as diuretics, beta-blockers, calcium-channel blockers, and angiotensin-converting enzyme or angiotensin II receptor blockers, are available currently, a systolic/diastolic blood pressure level of <140/90 mmHg, defined in the Seventh Report of the Joint National Committee (JNC7) in 2003 and reaffirmed in the JNC8 in 2013, was achieved in no more than 25 % of patients undergoing treatment for hypertension worldwide [5]. Furthermore, the clinical applications of antihypertensive drugs are limited by numerous adverse side effects, such as dizziness, headache, orthostatic hypotension, and decreased sexual function [6]. Notably, complementary and alternative therapies have become increasingly popular for treating hypertension [7], particularly traditional Chinese medicine (TCM) [8], because of their potential efficacy and few side effects [9, 10]. In addition to numerous published cases and randomized trials, an increasing number of experimental studies have revealed multiple antihypertensive and protective mechanisms of TCM [10]. In addition, various meta-analyses and systemic reviews have recently evaluated the effectiveness of TCM for hypertension [11].

TCM, including acupuncture, traumatology manipulative therapies, and decoction, plays a crucial role in health care in Taiwan and other Asian or Western countries. Chinese herbal products (CHPs), a modern form of decoctions in which herbal formulae and single herbs are concentrated into granulated compounds, are widely prescribed by TCM physicians because of their convenience and quality. According to TCM theory, TCM physicians evaluate patient conditions to prescribe one or more herbal formulae combined with several single herbs for each prescription. However, the CHPs used for treating hypertension and their co-prescription patterns remain not fully elucidated.

The National Health Insurance (NHI) program has been reimbursing claims for CHPs, including single herbs or herbal formulae, in Taiwan since 1995. By using the National Health Insurance Research Database (NHIRD), we analyzed the CHPs prescribed for treating hypertension and their co-prescription patterns in Taiwan. The results are a reference from which clinical practitioners can understand the medicinal demands and preferences of TCM users with hypertension and thereby provide reliable information about CHP use.

## Methods

### Data source

The NHI program of Taiwan, executed in 1995, reimburses both Western medicines and CHPs. Approximately 99 % of Taiwan residents were included into the program by the end of 2010 [12]. The electronic database of all claims obtained from the NHIRD website involved medical record files containing patient sex and date of birth, date of medical visits, medical care facilities and specialties, drugs, management and treatment, transferred identification number, and three major diagnoses coded according to the International Classification of Diseases, Ninth Revision, Clinical Modification (ICD-9-CM) format. The data regarding patient identity and institutions are cryptographically scrambled to protect personal privacy. Thus, the NHIRD offers an optimal platform for understanding the utilization pattern of CHPs prescribed by licensed TCM physicians among the hypertension patient population in Taiwan.

We analyzed a sample of one million randomly selected patients from among the 22 million beneficiaries of the NHI program in Taiwan, determining the prevalence of prescribed CHPs in patients with hypertension from 2003 to 2009. This study adhered to strict confidentiality guidelines according to regulations for personal electronic data protection and was approved by the Ethics Review Board of Chang Gung Memorial Hospital, Chia-Yi Branch (103-7691B).

### Study subjects

The study cohort comprised patients diagnosed with hypertension (ICD-9-CM codes: 401–405) three or more times within one year from 2003 to 2009; information was obtained from Western and TCM outpatient visit records. All hypertension-related medical records were analyzed during the study period. In the hypertension cohort, patients who had at least one TCM outpatient clinical visit from 2003 to 2009 were defined as TCM users; among the TCM users, those who had used CHPs were defined as CHP users, whereas those with no TCM outpatient records were defined as non-TCM users. In addition, we obtained the prescription files containing the CHP prescription records corresponding to patient outpatient visits that involved a hypertension diagnosis to assess the corresponding CHP co-prescriptions for hypertension.

### Study variables

Patient demographic characteristics were investigated to determine the main independent variables affecting TCM use in the hypertension cohort. In addition to patient sex, patient age was categorized into three groups: <45, 45–65, >65 years. The monthly insurance salaries of patients were grouped into four levels: NT$0, NT$1–NT$15,840, NT$15,841–NT$25,000, >NT$25,000. Urban levels in this study were divided into four strata according to the location of NHI registration. Level 1 referred to the most urbanization and level 4 indicated the least urbanization. We also included the following clinical, potential comorbidities related to hypertension from the NHIRD as independent variables: ischemic heart disease (ICD-9-CM codes: 410–414), cardiovascular disease (ICD-9-CM codes: 430–438), atherosclerosis (ICD-9-CM code: 440), hyperlipidemia (ICD-9-CM code: 272), and type II diabetes mellitus (ICD-9-CM code: 250).

### Traditional Chinese medicine

TCM, comprising CHPs, acupuncture, and traumatology manipulative therapies, has developed over the past millennia and is most widely adopted by patients in Taiwan. Prescriptions by TCM physicians based on the patients’ varying signs and symptoms may comprise one or more herbs (formulae). Chinese herbal drugs or formulae are manufactured as powder or fine granules that can be easily mixed in a single prescription. Information regarding reimbursed CHPs, including the name of each CHP, the proportion of each constituent, the date and period of drug approval, the code, and the manufacturer name, were obtained from the Department of Chinese Medicine and Pharmacy website.

### Statistical analysis

**Fig. 1:**
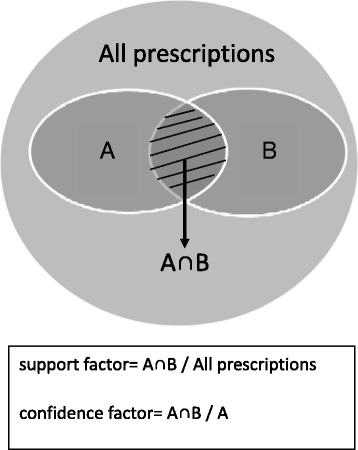
Basic Concepts and Algorithms of Association rules

Drug registration numbers from the Department of Chinese Medicine and Pharmacy website were linked to the outpatients visit records of the hypertension cohort. Database software, SAS Version 9.2 (SAS Institute Inc., Cary, NC, USA), was used for data linkage and descriptive statistic analysis of drug utilization pattern, including frequencies, percentages, average daily dose(g), and average use duration(days) of herbal formulae or single herbs for treating hypertension. In general, TCM physicians prescribe one or more herbal formulae including several single herbs to treat patients. In this study, the association rules of data mining were applied to assess the co-prescriptions of CHPs for hypertension. The support factor was the proportion of the co-prescriptions of medications A and B among all prescriptions, and the confidence factor was the proportion of the co-prescriptions of medications A and B among all prescriptions containing medication A (Fig. 1). We used 0.5 % as the minimum support factor and 30 % as the minimum confidence factor [13].

## Results

In this study, we identified 154,083 patients who had visited the outpatient department for hypertension three or more times within one year during the study period among the study cohort of one million randomly sampled patients. Table 1 shows a summary of the patient characteristics. Of these patients, 123,240 patients (approximately 80 %) used TCM at least once, and 116,070 patients used CHPs. Female patients used TCM more frequently than did male patients. The peak age of the patients receiving TCM treatment ranged between 45 to 65 years (47.4 %). In addition, patients with comorbidities such as metabolic diseases, including diabetes mellitus or hyperlipidemia, were more likely to consider TCM treatment.

**Table 1:** Characteristics of Hypertension population from one million random sampling cohort of the NHIR database

Characteristics	Non-TCM Users		TCM users		CHP users	
	No.	%	No.	%	No.	%
**Age**						
<45	3802	12.3	18768	15.2	17653	15.2
45-65	12790	41.5	58453	47.4	55219	47.6
>65	14251	46.2	46019	37.3	43198	37.2
**Gender**						
Female	11509	37.3	64826	52.6	61594	53.1
Male	19334	62.7	58414	47.4	54476	46.9
**Insurance salaries(NTD$/month)** ^**1**^						
0	5965	19.3	21891	17.8	20485	17.7
1-15840	5653	18.3	18185	14.8	16980	14.6
15841-25000	12882	41.8	57967	47.0	55006	47.4
>25000	6343	20.6	25197	20.4	23599	20.3
**Urban Level** ^**2**^						
1	9069	29.4	35622	28.9	33226	28.6
2	13023	42.2	55239	44.8	52063	44.9
3	5538	18.0	20546	16.7	19486	16.8
4	3213	10.4	11833	9.6	11295	9.7
**Comorbidity with hypertension(ICD-9-CM)**						
Ischemic Heart Disease (410–414)	9607	31.1	41603	33.8	39237	33.8
Cardiovascular disease (430–438)	7466	24.2	27218	22.1	25592	22.1
Atherosclerosis(440)	931	3.0	3814	3.1	3616	3.1
Hyperlipidemia (272)	11476	37.2	52627	42.7	49629	42.8
Type II Diabetes Mellitus(250)	9773	31.7	39936	32.4	37514	32.3
**Total**	30843		123240		116070	

1. NTD, New Taiwan dollar 1USD≒30NTD

2. 1:most urbanization; 4:least urbanization

Table 2 presents the estimated prevalence of hypertension (15.4 %) in Taiwan from 2003 to 2009, indicating that the prevalence increased with an increase in age for both sexes. The prevalence of hypertension was 2.5 %, 32.7 %, and 67.6 % in women and 4.1 %, 32.6 %, and 60.8 % in men aged <45, 45–65, and > 65 years, respectively.

**Table 2:** Patient number and estimated prevalence of hypertension in Taiwan from one million random sampling cohort of the NHIR database 2003-2009

Age/gender	All		Female		Male	
**All**	154083	15.4 %	76335	15.1 %	77748	15.7 %
**<45**	22570	3.3 %	8561	2.5 %	14009	4.1 %
**45-65**	71243	32.6 %	36066	32.7 %	35177	32.6 %
**>65**	60270	64.2 %	31708	67.6 %	28562	60.8 %

Between 2003 and 2009 in Taiwan, 81,582 hypertension-related outpatient visits involved CHP prescriptions by TCM physicians. Table 3 presents the most frequently prescribed herbal formulae and single herbs during these outpatient visits and includes data on the frequency of prescriptions, average daily doses, and average prescription durations. The most commonly prescribed herbal formula for hypertension was Tian-Ma-Gou-Teng-Yin (27.5 %), followed by Gou-Teng-San (12.5 %), Xue-Fu-Zhu-Yu-Tang (9.2 %), Jia-Wei-Xiao-Yao-San (7.8 %), and Zhi-Bo-Di-Huang-Wan (6.1 %). Moreover, Dan Shen *(Radix Salvia Miltiorrhizae)* (17.1 %) was the most commonly prescribed single herb for patients with hypertension, followed by Gou Teng *(Ramulus Uncariae cum Uncis)* (10.4 %), Ge Gen *(Radix Puerariae)* (8.4 %), and Niu Xi *(Radix Achyranthis Bidentatae)* (6.4 %).

**Table 3:** Top ten herbal formulae and single herb prescribed by traditional Chinese medicine doctors for Hypertension (n = 81,582)

	Frequency of prescription n (%)		Average daily dose (g)	Average duration for prescription (days)
**Herbal formulae**				
Tian-Ma-Gou-Teng-Yin	22,403	27.5 %	9.7	10.4
Gou-Teng-San	10,211	12.5 %	7.6	9.2
Xue-Fu- Zhu-Yu-Tang	7,540	9.2 %	7.7	10.7
Jia-Wei-Xiao-Yao-San	6,342	7.8 %	7.1	11.4
Zhi-Bo-Di-Huang-Wan	4,999	6.1 %	6.4	11.6
Qi-Ju-Di-Huang-Wan	4,828	5.9 %	4.9	12.7
Long-Dan-Xie-Gan-Tang	4,589	5.6 %	8.4	10.2
Liu-Wei-Di-Huang-Wan	4,058	5.0 %	6.8	11.0
Zhi-Gan-Cao-Tang	3,834	4.7 %	8.0	11.5
Shu-Jing-Huo-Xue-Tang	3,661	4.5 %	5.2	11.8
**Single Herb**				
Dan Shen *(Radix Salvia Miltiorrhizae)*	13,970	17.1 %	2.3	11.3
Gou Teng *(Ramulus Uncariae cum Uncis)*	8,492	10.4 %	2.0	10.4
Ge Gen *(Radix Puerariae)*	6,846	8.4 %	1.9	10.4
Niu Xi *(Radix Achyranthis Bidentatae)*	5,230	6.4 %	2.3	11.9
Xia Ku Cao *(Spica Prunellae Vulgaris)*	4,964	6.1 %	2.2	10.8
Da Huang *(Radix et Rhizoma Rhei)*	4,964	6.1 %	1.0	10.8
Du Zhong *(Cortex Eucommiae)*	4,576	5.6 %	1.7	11.9
Tian Ma *(Rhizoma Gastrodiae Elatae)*	4,284	5.3 %	1.7	10.9
Chuan Qi *(Radix Notoginseng)*	4,189	5.1 %	3.0	10.5
Shan Zha *(Crataegi Fructus)*	4,173	5.1 %	1.8	10.8

According to the association rules in Table 4, the most commonly prescribed combination of two herbal formulae for hypertension was Tian-Ma-Gou-Teng-Yin and Xue-Fu-Zhu-Yu-Tang (support factor, 2.64 %). The most commonly prescribed combination of a herbal formula and a single herb for treating hypertension was Tian-Ma-Gou-Teng-Yin and Dan Shen (support factor, 5.11 %). In addition, TCM physicians frequently prescribed Tian-Ma-Gou-Teng-Yin in combination with other herbal formulae, such as Xue-Fu-Zhu-Yu-Tang, Zhi-Bo-Di-Huang-Wan, Qi-Ju-Di-Huang-Wan, and Long-Dan-Xie-Gan-Tang, or with another single herb, such as Dan Shen, Ge Gen, and Xia Ku Cao *(Spica Prunellae Vulgaris)*. The most commonly prescribed combination of two single herbs was Tian Ma *(Rhizoma Gastrodiae Elatae)* and Gou Teng (support factor, 2.28 %). Moreover, physicians frequently prescribed Dan Shen in combination with other single herbs, such as Ge Gen, Chuan Qi *(Radix Notoginseng)*, Gou Teng, and Shan Zha *(Crataegi Fructus)* for treating hypertension.

**Table 4:** Co-prescriptions (one to one association) of single herbs and herbal formulae for Hypertension population

Herbal associations	Support (%)	Confidence (%)	Transaction count	Association rule
**Single herb to single herb**	2.28	43.46	1862	Tian Ma *(Rhizoma Gastrodiae Elatae)* → Gou Teng *(Ramulus Uncariae cum Uncis)*
	2.19	26.09	1785	Ge Gen *(Radix Puerariae)* → Dan Shen *(Radix Salvia Miltiorrhizae)*
	2.16	42.15	1764	Chuan Qi *(Radix Notoginseng)* → Dan Shen *(Radix Salvia Miltiorrhizae)*
	2.14	20.56	1745	Gou Teng *(Ramulus Uncariae cum Uncis)* → Dan Shen *(Radix Salvia Miltiorrhizae)*
	1.69	33.09	1380	Shan Zha *(Crataegi Fructus)* → Dan Shen *(Radix Salvia Miltiorrhizae)*
**Herbal formula to herbal formula**	2.64	28.55	2151	Xue-Fu- Zhu-Yu-Tang → Tian-Ma-Gou-Teng-Yin
	1.89	30.77	1538	Zhi-Bo-Di-Huang-Wan → Tian-Ma-Gou-Teng-Yin
	1.82	30.91	1486	Qi-Ju-Di-Huang-Wan → Tian-Ma-Gou-Teng-Yin
	1.60	28.49	1307	Long-Dan-Xie-Gan-Tang → Tian-Ma-Gou-Teng-Yin
**Herbal formula to single herb**	5.11	18.61	4166	Tian-Ma-Gou-Teng-Yin → Dan Shen *(Radix Salvia Miltiorrhizae)*
	2.66	9.68	2168	Tian-Ma-Gou-Teng-Yin → Ge Gen *(Radix Puerariae)*
	2.47	9.00	2014	Tian-Ma-Gou-Teng-Yin → Xia Ku Cao *(Spica Prunellae Vulgaris)*
	2.02	21.83	1645	Xue-Fu- Zhu-Yu-Tang → Dan Shen *(Radix Salvia Miltiorrhizae)*
	1.64	5.99	1340	Tian-Ma-Gou-Teng-Yin → Du Zhong *(Cortex Eucommiae)*

## Discussion

To the best of our knowledge, this is the first large, random national-level study to document the frequency and co-prescription pattern of CHPs, including herbal formulae and single herbs, for hypertension. The results were obtained by analyzing the computerized insurance reimbursements in Taiwan. The term “hypertension” did not exist in ancient China; thus, TCM has been used to treat hypertension-related symptoms in clinical practice rather than to decrease blood pressure values. In other words, TCM physicians treat patients according to a holistic consideration of the body’s condition. The most common clinical manifestations of hypertension include dizziness, headache, fatigue, shortness of breath, lassitude in the loins and knees, memory loss, dry eyes, and palpitations [14]. TCM physicians prescribe various Chinese herbal formulae or single herbs to treat such patients by differentiating the syndromes according to the signs and symptoms caused by hypertension and TCM principles.

Because of the unsatisfactory efficacy and potential side effects of antihypertensive drugs, including dizziness, headache, orthostatic hypotension, and decreased sexual function after long term use [6],the use of TCM as an alternative therapeutic option for hypertension has been increasing in Taiwan. Although several studies on CHPs have reported their effectiveness in treating hypertension [7, 8, 15], additional studies are warranted to further investigate the safety and efficacy of CHPs and their drug interactions.

Tian-Ma-Gou-Teng-Yin, the most commonly prescribed herbal formula for hypertension in Taiwan, as reported by a previous study [16], has been demonstrated to lower blood pressure and total cholesterol, prevent the incidence of stroke in patients with hypertension, and improve clinical symptoms and quality of life [17, 18]. Several clinical studies and animal experiments have revealed that Tian-Ma-Gou-Teng-Yin decreases endothelin, angiotensin II [19], superoxide dismutase [20],and calcium gene-related peptides [21], regulates the secretions and serum concentrations of vasoactive substances [22], and enhances insulin resistance [23]. However, a review article reported that no randomized, controlled clinical trials have compared the effects of Tian-Ma-Gou-Teng-Yin with those of a placebo or no treatment. Thus, further research is warranted to establish the efficacy of Tian-Ma-Gou-Teng-Yin [24].

Gou-Teng-San is the second most commonly prescribed herbal formula for hypertension. Gou-Teng-San is often prescribed for headache and vertigo and has recently been prescribed for treating hypertension and dementia [25]. Reportedly, the antihypertensive effect of Gou-Teng-San is mediated by calcium channel antagonist action, which was demonstrated clearly in the pharmacological analysis of hirsutine, an indole alkaloid isolated from Gou Teng [26]. The other possible mechanism for the antihypertensive effect of Gou-Teng-San is the radical scavenging activity of Gou Teng, a main component herb of Gou-Teng-San, in the isolated aorta with the endothelium, reported in an experimental animal model [27].

Xue-Fu-Zhu-Yu-Tang, the third most commonly prescribed herbal formula for hypertension, is commonly prescribed for cardiovascular diseases by TCM physicians in clinical practice. Patients with hypertension typically exhibit enhanced platelet adhesion, aggregation, releasing reaction, and dysfunction in erythrocyte deformability; hence, improving erythrocyte deformability and inhibiting platelet activation might be helpful in treating patients with hypertension. The use of CHPs might improve blood rheological conditions, including coagulation, viscosity, blood flow, and deformability [10]. Certain experimental studies have demonstrated that Xue-Fu-Zhu-Yu-Tang can increase coronary blood flow, improve cardiac microcirculation, prevent platelet aggregation, and accommodate blood lipids [28, 29].

Although Jia-Wei-Xiao-Yao-San is rarely prescribed alone by TCM physicians for treating hypertension, this study revealed that it was the fourth most commonly prescribed herbal formula for hypertension. TCM physicians combined it with another herbal formula and/or single herb according to patient symptoms [30]. Moreover, previous studies have reported that Jia-Wei-Xiao-Yao-San is the most commonly used CHP for relieving menopausal symptoms, such as hot flushes, and other related symptoms, including emotional disturbances and insomnia [31, 32]. Patients with such symptoms might be at a higher risk of developing hypertension, and this may explain the frequent prescription of Jia-Wei-Xiao-Yao-San for hypertension.

As observed in Table 3, Dan Shen, the dried root and the rhizome of *Salviamiltiorrhiza Bge* (Labiatae), is the most frequently prescribed single herb for hypertension. Studies have reported the multiple pharmacological activities of Dan Shen in the cardiovascular system, including anti-hypertension [33], anti-thrombosis [34], anti-atherosclerosis [35],and cardioprotection [36], in addition to improved microcirculation in the brain and heart [37]. Previous studies have demonstrated that the active chemical constituents of Dan Shen, particularly, tanshinone IIA, danshensu, salvianolic acid B, lithospermicacids, and cryptotanshinone, exhibit beneficial effects in managing cardiovascular diseases [38, 39]. For over five decades, using Dan Shen products has been considered extremely safe, with no reports of any major adverse effect [40]. Although studies have reported its interactions with warfarin, salicylate, diazepam, and ginseng [41–44], no studies have reported interactions between antihypertensive drugs and Dan Shen.

Gou Teng is the second most frequently prescribed single herb for hypertension. Certain studies have reported that alkaloids are the main active pharmacological components of Gou Teng. Of these components, rhynchophylline and isorhynchophylline are the chief ingredients, which play a major role as hypotensive agents [45]. As observed in Table 4, TCM physicians often used Tian Ma in combination with Gou Teng for treating hypertension or hypertension-related symptoms. Tian Ma is also a crucial Chinese herb used for decreasing blood pressure values. Moreover, recent studies have demonstrated that it can promote a stable decrease in blood pressure, improve clinical symptoms, regulate the rennin–angiotensin system, improve insulin resistance and salt sensitivity, and decrease serum levels of total cholesterol, triglycerides, and low-density lipoprotein cholesterol [15, 46, 47].

To increase treatment efficacy and compensate for insufficiency of another single herb, TCM physicians often use two single herbs as a herb pair. Tian Ma used with Gou Teng is an example of a herb pair that increases treatment efficacy and exerts similar effects in the cardiovascular and central nervous systems. This might explain why Tian Ma and Gou Teng are the two most frequently prescribed single herbs in combination for treating hypertension.

In addition, TCM physicians often used Ge Gen to treat hypertension-related symptoms such as headache and dizziness. Previous studies have reported that Ge Gen primarily comprises isoflavonoids, such as puerarin, diadzein, and daidzin, which exhibit anti-hypertensive [48] and antithrombotic effects [49] and reduce plasma cholesterol levels [50].

TCM physicians treat patients with hypertension according to syndrome differentiation theory, and prescriptions differed among TCM physicians because of their experience and knowledge. In the present study, the co-prescription patterns of CHPs by TCM physicians differed from that of decoctions. TCM physicians prescribed a thoroughly recorded formula as the core formula and subtracted or added herbs in a decoction preparation to fit a patient’s condition according to TCM theory. When they prescribed CHPs, which are extracted and completely mixed together in the manufacturing process, TCM physicians only added single or multiple herbs into the core formula, but subtracted or changed the proportions of herbs in the finished herbal products. Thus, TCM physicians combined other formulae or herbs in each prescription to treat patients. Moreover, the NHI in Taiwan only reimburses CHPs and not decoctions, eliciting our concern that the co-prescription pattern of CHPs with a high support factor might indicate a typical practice pattern among TCM physicians in Taiwan [51]. Although 5.9 CHPs were prescribed on average during one hypertension-related TCM outpatient visit in the present study, the co-prescription of three or more Chinese herbal formulae or single herbs for hypertension had a low support factor; thus, we determined two co-prescription patterns (one to one association) in the study.

Tian-Ma-Gou-Teng-Yin and Xue-Fu-Zhu-Yu-Tang was the most commonly prescribed combination of two herbal formulae for treating hypertension, whereas Tian-Ma-Gou-Teng-Yin and Dan Shen were the most frequently prescribed combination of an herbal formula and a single herb for treating hypertension. In addition, Tian-Ma-Gou-Teng-Yin and Dan Shen were the most frequently co-prescribed herbal formula and single herb for treating hypertension. Furthermore, we could investigate the most common corresponding diagnoses or syndromes related to hypertension. As in a previous study [32], we can conduct clinical trials on the efficacy and safety of these co-prescribed CHPs according to the results of our study in the future.

Although patients with hypertension primarily seek conventional therapy, TCM is crucial in complementary and alternative medicines. In other words, most patients with hypertension who went to TCM outpatient clinical practices for treatment often had an initial hypertension diagnosis from Western medicine practitioners. Furthermore, modern TCM physicians are trained in Western medicine knowledge during undergraduate or refresher courses. Although, the therapeutic principles of TCM are based on the results of syndrome differentiation, which differs from the principles of Western medicine, TCM physicians in Taiwan must use ICD-9-CM codes in the NHI claims database to diagnose diseases during outpatient visits.

A previous study revealed that some patients seek TCM treatment for problems other than hypertension, whereas some patients were treated for hypertension with acupuncture and traumatology manipulative therapies [16]. This might explain why only 81,582 outpatient hypertension-related visits involved CHP prescriptions by TCM physicians among the 123,240 TCM visits investigated in the present study. The prevalence of hypertension increases with age, especially when people are older than 65 years. These findings were consistent with those of a previous study [52]. However, our cohort included patients who had visited the outpatient department because of hypertension three or more times within one year alone during the study period of 2003–2009; this might have led to an underestimation of the prevalence of hypertension in Taiwan. In the present study cohort, women and patients aged 45–65 years used TCM more frequently than did men and patients in the other age groups. In addition, the patients with hypertension who experienced additional comorbidities such as metabolic diseases were more likely to consider TCM treatments.

This study had three limitations. First, we could not obtain definitive conclusions regarding the relationship between the severity of hypertension and TCM utilization. Second, apparently, TCM physicians in Taiwan use ICD-9-CM for diagnosis in clinical practice, but no reliable and suitable disease coding system exists for TCM. Several variations were observed in the prescription patterns for treating hypertension because the therapeutic principles of TCM are based on the results of syndrome differentiation. Thus, developing a coding system for TCM diagnostic classifications in the future is critical and will assist TCM research greatly. Third, NHI provided reimbursements for only finished herbal products prescribed by TCM physicians and did not include decoctions and CHPs provided by pharmacies; this might have led to the underestimation of the frequency of TCM utilization. However, this underestimation might be small because most CHPs were reimbursed.

## Conclusions

This study analyzed a cohort of one million randomly sampled patients from the NHIRD from 2003 to 2009 and documented the frequency and co-prescription pattern of CHPs for hypertension in clinical practice in Taiwan. However, further research must be conducted to strengthen the available clinical evidence regarding the efficacy and safety of CHPs in improving hypertension-related mortality and morbidity as well as the drug–herb interactions.

## Abbreviations

CHPs: Chinese herbal products
TCM: Traditional Chinese Medicine
NHI: National Health Insurance
NHIRD: National Health Insurance Research Database
ICD-9-CM: International Classification of Diseases, Ninth Revision, Clinical Modification
